# Improved conversion of ginsenoside Rb_1_ to compound K by semi-rational design of *Sulfolobus solfataricus* β-glycosidase

**DOI:** 10.1186/s13568-017-0487-x

**Published:** 2017-10-04

**Authors:** Kyung-Chul Shin, Hye-Yeon Choi, Min-Ju Seo, Deok-Kun Oh

**Affiliations:** Department of Bioscience and Biotechnology, Konkuk University, Seoul, 05029 Republic of Korea

**Keywords:** Ginsenoside compound K, *Sulfolobus solfataricus* β-glycosidase, Semi-rational design

## Abstract

Ginsenoside compound K has been used as a key nutritional and cosmetic component because of its anti-fatigue and skin anti-aging effects. β-Glycosidase from *Sulfolobus solfataricus* (SS-BGL) is known as the most efficient enzyme for compound K production. The hydrolytic pathway from ginsenoside Rb_1_ to compound K via Rd and F_2_ is the most important because Rb_1_ is the most abundant component in ginseng extract. However, the enzymatic conversion of ginsenoside Rd to F_2_ is a limiting step in the hydrolytic pathway because of the relatively low activity for Rd. A V209 residue obtained from error-prone PCR was related to Rd-hydrolyzing activity, and a docking pose showing an interaction with Val209 was selected from numerous docking poses. W361F was obtained by rational design using the docking pose that exhibited 4.2-fold higher activity, 3.7-fold higher catalytic efficiency, and 3.1-fold lower binding energy for Rd than the wild-type enzyme, indicating that W361F compensated for the limiting step. W361F completely converted Rb_1_ to compound K with a productivity of 843 mg l^−1^ h^−1^ in 80 min, and showed also 7.4-fold higher activity for the flavanone, hesperidin, than the wild-type enzyme. Therefore, the W361F variant SS-BGL can be useful for hydrolysis of other glycosides as well as compound K production from Rb_1_, and semi-rational design is a useful tool for enhancing hydrolytic activity of β-glycosidase.

**Electronic supplementary material:**

The online version of this article (doi:10.1186/s13568-017-0487-x) contains supplementary material, which is available to authorized users.

## Introduction

**Fig. 1:**
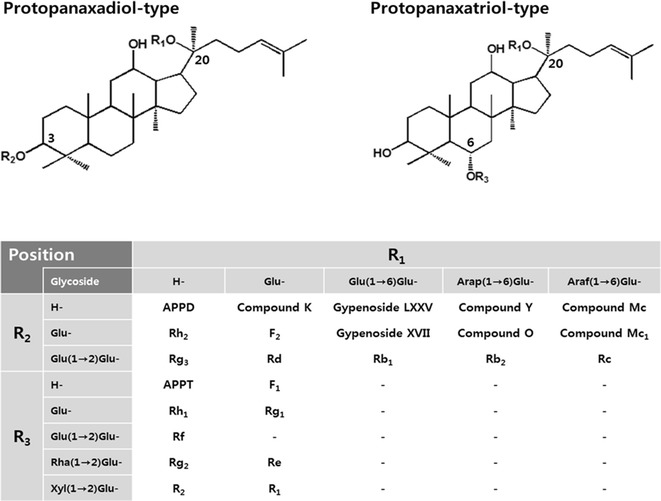
Chemical structures of protopanaxadiol (PPD) and protopanaxatriol (PPT)-type ginsenosides. *Glu* β-d-glucopyranosyl, *Arap* α-l-arabinopyranosyl, *Araf* α-l-arabinofuranosyl, *Rha*, α-l-rhamnopyranosyl, *Xyl*, β-d-xylopyranosyl

Ginsenosides, which are the active components of the valuable herb ginseng (*Panax ginseng* C. A. Meyer), have been used as traditional herbal medicines in Asian countries (Lu et al. 2009). They have diverse beneficial biological properties such as anti-cancer (Lee et al. 2009), anti-oxidant (Cho et al. 2006), anti-inflammatory (Wang et al. 2012), anti-allergic (Bae et al. 2002), anti-fatigue (Yoshikawa et al. 2003), and anti-skin aging (Kang et al. 2009) activities. Most ginsenosides are classified into two types: a protopanaxadiol (PPD)-and protopanaxatriol (PPT)-types harboring 1–4 molecule glycosides such as d-glucose, l-arabinosepyranose, and l-arabinofuranose at C-3 and/or C-20, and d-glucose, l-rhamnose and d-xylose at C-6 and/or C-20 (Fig. 1), and the abbreviations of ginsenosides are shown in Additional file 1: Table S1. Glycosylated ginsenosides (Rb_1_, Rb_2_, Rc, Rd, Rg_1_, and Re) constitute more than 80% of the total ginsenosides as major ginsenosides in wild ginseng (Park et al. 2010; Son et al. 2008), however, minor deglycosylated ginsenosides (F_2_, Rg_3_, Rh_1_, Rh_2_, and compound K) exhibit superior biological activity compared to major glycosylated ginsenosides because they possess smaller-sized structures, higher bioavailability, and better permeability across the cell membrane (Kim et al. 2005).

Compound K, one of the most pharmaceutically active PPD-type ginsenosides, has gained attention in recent years because it is effective for the inhibition of tumor invasion (Wakabayashi et al. 1998), shows hepatoprotective activity (Lee et al. 2005), induces tumor cell apoptosis (Oh et al. 2004), and prevents wrinkling and skin damage (Lim et al. 2015; Shin et al. 2014). Because compound K is absent in wild ginseng, it has been produced from ginseng extract by enzymatic reactions using recombinant β-glycosidases (Liu et al. 2014; Noh and Oh 2009; Quan et al. 2012, 2013; Yan et al. 2008; Yu et al. 2007). Of these enzymes, *Sulfolobus solfataricus* β-glycosidase (SS-BGL) is the most efficient enzyme for the production of compound K from ginseng extract because it has broad substrate specificity and high activity for ginsenosides (Noh et al. 2009; Shin et al. 2015). The hydrolysis of ginsenoside Rb_1_ to compound K via Rd and F_2_ as intermediates is the most important among the hydrolyses of major PPD-type ginsenosides by SS-BGL because ginsenoside Rb_1_ is the most abundant component in ginseng extract (Ji et al. 2001). However, the enzymatic conversion of ginsenoside Rd to F_2_ is a limiting step in the hydrolytic pathway because SS-BGL exhibits approximately 15-fold lower activity for ginsenoside Rd than Rb_1_. For the effective production of compound K, a variant SS-BGL that shows increased activity for ginsenoside Rd is needed.

In the field of protein engineering, rational design and directed evolution have been used for obtaining efficient biocatalysts. However, directed evolution requires a high-throughput screening system and a large number of libraries, and rational design requires detailed and accurate structural knowledge of a protein. To address these limitations, a combined approach has been proposed as semi-rational design (Chen et al. 2012; Lutz 2010). In this approach, rational design is based on the variant biocatalysts obtained from a small library than the traditional random mutagenesis library, and thus, does not require high-throughput screening.

In the present study, we found that the V209 residue was related to Rd-hydrolyzing activity by analysis of variant enzymes constructed from random mutagenesis using error-prone PCR. A W361F variant that showed higher hydrolytic activity for ginsenoside Rd than the wild-type SS-BGL was obtained by rational design using the docking pose interacting with Val209 among the ginsenoside Rd-docking SS-BGL models. The substrate specificity, kinetics parameters, and compound K production from ginsenoside Rb_1_ of the variant enzyme were investigated and compared with those of the wild-type enzyme.

## Materials and methods

### Microorganisms, medium, and culture conditions

*Sulfolobus solfataricus* DSM 1617, *Escherichia coli* ER2566, and plasmid pET-24a were used as the sources of the β-glycosidase, host cells, and expression vector, respectively. To express β-glycosidase, recombinant *E. coli* cells were cultivated in a 2-l flask containing 500 ml of Luria-Bertani (LB) medium supplemented with 20 μg ml^−1^ of kanamycin at 37 °C with agitation at 200 rpm. When the optical density of the bacterial culture at 600 nm reached 0.6, isopropyl-β-d-thiogalactopyranoside (IPTG) was added to the culture to a final concentration of 1.0 mM to induce enzyme expression. After induction, the cells were further incubated with shaking at 150 rpm at 16 °C for 16 h.

### Enzyme purification

The harvested *E. coli* cells were resuspended in 50 mM citrate/phosphate buffer at pH 5.5 and pH 7.0 containing 300 mM NaCl. The resuspended cells were disrupted by sonication using a Sonic Dismembrator (Fisher Scientific Model 100, Pittsburgh, PA, USA) on ice for 10 min. The unbroken cells and cell debris were removed by centrifugation at 13,000×*g* for 20 min at 4 °C, and the supernatant was used as a crude extract. The crude extract in 50 mM citrate/phosphate buffer (pH 5.5) was subsequently heated at 75 °C for 10 min. After heat treatment, the suspension was centrifuged at 13,000×*g* for 20 min to remove insoluble denatured proteins. The supernatant obtained was used as the partially purified enzyme. The crude extract in 50 mM phosphate buffer (pH 7.0) was applied to a His-trap HP affinity chromatography column (GE Healthcare, Piscataway, NJ, USA). The bound protein was eluted with a linear gradient of 10–250 mM imidazole with 50 mM phosphate buffer (pH 7.0) at a flow rate of 1 ml min^−1^. The active fractions were collected and dialyzed against 50 mM citrate/phosphate buffer (pH 5.5) at 4 °C for 16 h. The resulting solution was used as the purified enzyme. The purification step with the column was carried out in a cold room at 4 °C with a fast protein liquid chromatography system (Bio-Rad, Hercules, CA, USA).

### Mutation of SS-BGL

SS-BGL was cloned and expressed in *E. coli* as previously described (Kim et al. 2006). For mutation of β-glycosidase genes, an error-prone PCR was conducted using a PCR mutagenesis kit (ClonTech Laboratories, Palo Alto, CA, USA). The mutated β-glycosidase genes were used as templates and sub-cloned into pET-24a using an assembly method (Gibson et al. 2009). Forward (5′-GTACCTCCAGTAAAGCCATTAAGGCACTAACTCGAGCACCACCACCACCACCACTGAGAT-3′) and reverse primers (5′-AAACCTAAAGCTATTTGGAAATGAGTACATCATATGTATATCTCCTTCTTAAAGTTAAAC-3′), and forward (5′-GTTTAACTTTAAGAAGGAGATATACATATGATGTACTCATTTCCAAATAGCTTTAGGTTT-3′) reverse primers (5′-ATCTCAGTGGTGGTGGTGGTGGTGCTCGAGTTAGTGCCTTAATGGCTTTACTGGAGGTAC-3′) were designed to amplify the expression vector and mutated β-glycosidase DNA fragments, respectively. The amplified linearized vector and DNA fragments were ligated using Gibson Assembly Master Mix (New England Biolabs), and the ligated DNA products were transformed into *E. coli* ER2566. Site-directed mutagenesis (SDM) was performed using a QuickChange kit and the manufacturer’s protocol (Stratagene, Beverly, MA, USA).

### Screening

Colonies of recombinant *E. coli* were grown on LB agar containing 20 μg ml^−1^ kanamycin and were transferred to a 96-well plate containing 50 μl LB medium supplemented with 20 μg ml^−1^ kanamycin. The 96-well plate was incubated at 37 °C with shaking at 200 rpm for 3 h. Subsequently, 50 μl of 50 mM citrate/phosphate buffer (pH 5.5) containing p-nitrophenol (pNP)-β-d-glucopyranoside (2 mM) as a substrate was added to the 96-well plate, the plate was incubated at 90 °C for 10 min to allow for cell lysis and hydrolytic reactions, and the reactions were then stopped by the addition of Na_2_CO_3_ at final concentration of 200 mM. The increase in absorbance at 405 nm was measured by microplate reader (BioTek Instrument, Seoul, Republic of Korea) to analyze the release of pNP. The selected colonies were incubated, and enzyme expression in cells was induced by adding IPTG as described in the culture conditions section. The variant enzymes were partially purified with heat treatment as described above. The reactions were performed in 50 mM citrate/phosphate buffer (pH 5.5) containing 0.001 mg ml^−1^ partially purified enzyme and 1 mM pNP-β-d-glucopyranoside at 90 °C for 10 min. The selected variant enzymes were purified by His-trap chromatography as described above. The reactions were performed in 50 mM citrate/phosphate buffer (pH 5.5) containing 0.02 mg ml^−1^ purified enzyme and 0.5 mM ginsenoside Rd at 90 °C for 10 min.

### Ligand docking

Homology modeling of the wild-type and W361F variant SS-BGLs was performed using the Build Homology Models module in the MODELER application of Discovery Studio (DS) 4.0 (Accerlys, San Diego, CA) based on the crystal structure of SS-BGL [Protein data bank (PDB) entry, 1GOW] as a template. Comparative modeling was used to generate the most probable structure of the queried protein by aligning it with the template sequence, simultaneously considering spatial restraints, and local molecular geometry. The quality of the model was analyzed by PROCHECK (Laskowski et al. 1993). Hydrogen atoms were added to the model and minimized to have a stable energy conformation and to relax the conformation from close contacts. Ginsenoside Rd as a substrate was docked into the active-site pocket in the model of the wild-type and W361F variant SS-BGLs using the C-DOCKER module, and the active site was derived from a structure of 1GOW. Candidate poses were created using random rigid-body rotations, followed by simulated annealing. The structures of the protein, substrate, and their complexes were subjected to energy minimization using the CHARMM force field in DS 4.0 (Al-Balas et al. 2012). Full-potential final minimization was used to refine the substrate poses. The energy-docked conformation of the substrate was retrieved for postdocking analysis using the C-DOCKER module. The substrate orientation giving the lowest interaction energy was chosen for subsequent rounds of docking. The binding energy between receptor and ligand (*∆E*_Binding_) were defined as *E*_Complex_ − *E*_Ligand_ − *E*_Receptor_ (Tirado-Rives and Jorgensen 2006).

### Enzyme reactions and determination of kinetic parameters

Unless otherwise stated, the reactions were performed at 95 °C for 10 min in 50 mM citrate/phosphate buffer (pH 5.5) containing 1 mM aryl-glycoside and 0.001 mg ml^−1^ enzyme or 0.5 mM ginsenoside and 0.02 mg ml^−1^ enzyme. The aryl-glycoside activity was determined by measuring the increase in absorbance at 405 nm due to the release of pNP. The effects of pH and temperature on the activities of the wild-type and W3621F variant enzymes for pNP-β-d-glucopyranoside were investigated by varying the pH from 4.0 to 7.0 using 50 mM citrate/phosphate buffer at 95 °C and by varying the temperature from 75 to 95 °C in 50 mM citrate/phosphate buffer (pH 5.5). Temperature was varied below 100 °C because the boiling temperature of water, 100 °C, was not accurately maintained. The hydrolytic reactions for ginsenosides and flavanone glycosides were performed in 50 mM citrate/phosphate buffer (pH 5.5) containing 0.05 mg ml^−1^ enzyme and 0.4 mM substrate at 95 °C for 5–20 min. The time-course reaction by the wild-type or W361F variant enzymes was performed at 95 °C in 50 mM citrate/phosphate buffer (pH 5.5) containing 2 mg ml^−1^ ginsenoside Rb_1_ and 0.01 or 0.1 mg ml^−1^ enzyme for 60 or 90 min, respectively.

To determine the kinetic parameters of the wild-type and W361F variant enzymes, the concentrations of ginsenosides Rb_1_ and Rd substrates used were in the ranges of 0.125–2 mM. The *K*_m_ and *k*_cat_ for the substrates were calculated by a Hans-Woolf plot from the Michaelis–Menten equation.

### Analytical methods

A reaction solution containing digoxin as an internal standard and ginsenosides as substrates and products was extracted by adding an equal volume of n-butanol. The solvent in the extracted solution was evaporated and methanol was added to the dried sample. Ginsenosides were assayed using an HPLC system (1100, Agilent, Santa Clara, CA, USA) equipped with a UV detector at a wavelength of 203 nm using a C18 column (YMC, Kyoto, Japan). The column was eluted with a linear gradient of acetonitrile/waster from 20:80 to 80:20 (v/v) and a flow rate of 1 ml min^−1^ for 80 min at 37 °C. Ginsenoside concentrations were determined using linear calibration curves relating the peak areas to the ginsenoside standard concentrations.

### Statistical analyses

The means and standard errors for all experiments were quantitatively calculated with one-way analysis of variance (ANOVA) from triplicates. ANOVA was carried out using Tukey’s method with a significance level of *p* < 0.05 using SigmaPlot 10.0 (Systat Software, Chicago, IL).

## Results

### Determination of a residue related to ginsenoside Rd-hydrolyzing activity by screening of mutant library produced from error-prone

The hydrolytic activity of pNP-β-d-glucopyranoside is determined using a microplate reader, while ginsenoside Rd is determined using an HPLC system, indicating that the assay of pNP-β-d-glucopyranoside is more convenient and rapid. Thus, in the first and second rounds of selection, the hydrolytic activity was measured using pNP-β-d-glucopyranoside as a substrate. However, the increase in the hydrolytic activity for pNP-β-d-glucopyranoside does not mean exactly the increase in the hydrolytic activity for ginsenoside Rd. Therefore, in the final round, the hydrolytic activity was measured using ginsenoside Rd as a substrate.

A library of mutated β-glycosidase genes was constructed using an error-prone PCR with a mutation rate of 1–2 mutations per 1000 bp. For the first round of selection, cells were isolated and incubated from the initial 10,000 mutant colonies, and 500 cells expressing variant enzyme, which exhibited 1.2-fold greater hydrolytic activities for pNP-β-d-glucopyranoside than that of the wild-type cells, were selected. For the second round of selection, the cells selected at the first round were incubated, and β-glycosidases in cells were expressed. The β-glycosidases expressed in the crude extracts obtained from the culture broths of the selected cells were partially purified by heat treatment. The hydrolytic activities of the partially purified enzymes for pNP-β-d-glucopyranoside were estimated, and then 50 variant β-glycosidases showing 1.2-fold greater activity than that of the wild-type enzyme were selected. For the third round of selection, the heat-treated variant enzymes selected at the second round were purified by His-Trap chromatography, and the hydrolytic activities of the purified enzymes for ginsenoside Rd were determined. Ginsenosides were not hydrolyzed when the reactions were run under the experimental conditions without enzyme or with grown cells of *E. coli* ER2566, which did not contain the β-glycosidase gene from *S. solfataricus*. A V209A variant β-glycosidase, which showed 1.7-fold higher activity than the wild-type enzyme, was selected for an enzyme with the highest activity for the conversion of ginsenoside Rd to compound K. The finally selected variant enzyme and its DNA sequence were used for further studies such as structural analysis and SDM.

**Fig. 2:**
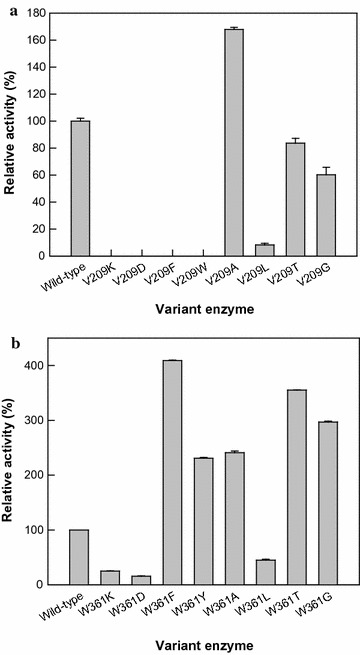
Activities of the variant SS-BGLs for ginsenoside Rd for the conversion to compound K. **a** Activities of the variant enzymes with mutations at position 209 of SS-BGL. **b** Activities of the variant enzymes with mutations at position 361 of SS-BGL. Data represent the means from three separate experiments, and the error bars represent standard deviations

The amino acid at position 209 in SS-BGL was replaced with a residue such as Lys, Asp, Phe, Trp, Gly, Ala, Leu, or Thr. The variant enzymes, V209G, V209A, V209T, and V209L exhibited hydrolytic activity for ginsenoside Rd, whereas V209F, V209 W, V209 K, and V209D exhibited no activity (Fig. 2a). Among the variant enzymes, V209A showed the highest activity for Rd.

### Acquisition of a variant with increased ginsenoside Rd-hydrolyzing activity by rational design based on the selected residue

**Fig. 3:**
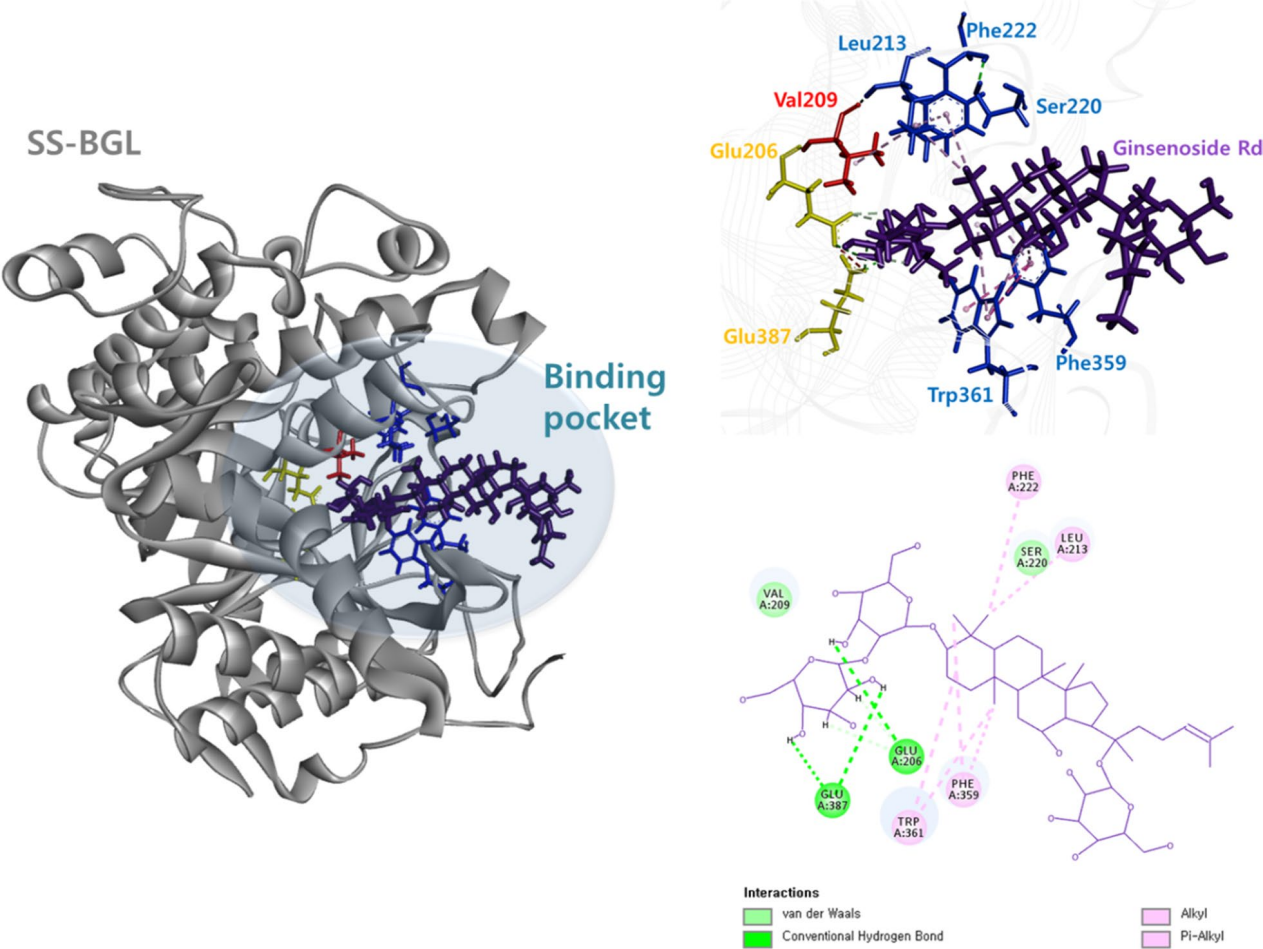
Docking pose for selection of candidate residues related to ginsenoside Rd-hydrolyzing activity. 3D- and 2D-structures represent receptor-ligand and receptor–receptor interaction, and only receptor-ligand interaction, respectively. SS-BGL (PDB entry, 1GOW), *grey* ginsenoside Rd, *purple* catalytic residues, *yellow* residues related to Rd-hydrolyzing activity, *red* candidate residues related to Rd-hydrolyzing activity, *blue* hydrophobic interactions, *pink dotted line* and hydrogen bonds, green dotted line

To obtain a variant with higher ginsenoside Rd-hydrolyzing activity than that of the V209A variant, ginsenoside Rd as a ligand was docked to the active site of SS-BGL [Protein data bank (PDB) entry, 1GOW, 2CEQ, and 2CER] (Aguilar et al. 1997; Gloster et al. 2006) as a receptor. Ginsenoside Rd was docked only to 1GOW but not docked to 2CEQ or 2CER. In the docking pose using 1GOW with ginsenoside Rd, Val209 interacted with the catalytic residue Glu206 and Leu213, ginsenoside Rd interacted with Leu213 and Phe222, and Phe222 interacted with Ser220 (Fig. 3). Thus, the residues Leu213, Phe222, and Ser220 were selected as putative candidate residues related to ginsenoside Rd-hydrolyzing activity. These residues indicated only hydrophobic interaction with ginsenoside Rd, whereas the catalytic residue interacted only with hydrogen bond. Therefore, two residues Phe359 and Trp361 that have two hydrophobic interactions with ginsenoside Rd were additionally selected as candidates.

These five residues were replaced with Ala, and the specific activities of the wild-type and Ala-substituted variant enzymes were determined using ginsenoside Rd as a substrate. The activities of L213A, S220A, F222A, and F359A, were 110, 92, 105, and 84% of the wild-type enzyme activity, respectively, whereas the activity of W361A was 241% of the wild-type enzyme activity, which was 1.4-fold higher than that of V209A. Thus, the W361A variant was the most efficient for ginsenoside Rd hydrolysis.

The Trp residue at position 361 of SS-BGL was substituted with Lys, Asp, Phe, Tyr, Gly, Ala, Leu, or Thr. The hydrolytic activity of the variant enzymes for ginsenoside Rd followed the order W361F > W361T > W361G > W361A > W361Y > wild-type > W361L > W361 K > W361D (Fig. 2b). The W361F variant enzyme showed the highest activity for ginsenoside Rd. Therefore, the W361F variant was selected for further investigations of its biochemical properties and the conversion of ginsenoside Rb_1_ to compound K.

### Binding energy of the wild-type and W361F variant enzymes

Homology modeling of the W361F variant SS-BGL was performed based on the crystal structure and binding energy of the variant enzyme was compared with that of wild-type enzyme. The binding energy (*∆E*_binding_) of the W361F variant enzyme docked with Rd (− 183.2 kcal mol^−1^) was lower than that with the wild-type enzyme (− 59.7 kcal mol^−1^). The C-DOCKER interaction energy, indicating the non-bonded energy between the ligand and the enzyme, of the W361F variant enzyme (− 87.6 kcal mol^−1^) was lower than the wild-type enzyme (− 65.8 kcal mol^−1^).

### Substrate specificity of the wild-type and W361F variant enzymes

The effects of pH and temperature on the activity of the W361F variant enzyme for pNP-β-d-glucopyranoside were compared with those of the wild-type enzyme. The maximum activities of both enzymes were observed at pH 5.5 and 95 °C (Additional file 1: Figure S1). Although the hydrolytic activity of the W361F variant enzyme for pNP-β-d-glucopyranoside was lower than that of the wild-type enzyme, the order of the hydrolytic activity for specific aryl-glycoside types of both enzymes were the same (Additional file 1: Table S2).

The activity of the W361F variant enzyme for F_2_ was similar to that of the wild-type enzyme, however, the substrate specificity for other PPD-type ginsenosides was distinctly different (Table 1). The specific activity of the W361F variant enzyme followed the order F_2_ > Rb_2_ > Rb_1_ > compound O > Rd > compound Y > Rc > compound Mc_1_ > compound Mc, while that of the wild-type enzyme followed the order F_2_ > compound O > Rb_1_ > compound Y > Rb_2_ > Rd > compound Mc_1_ > Rc > compound Mc.

**Table 1:** Specific activities of the wild-type and W361F variant enzymes for ginsenosides and flavanones

Substrate	Specific activity (nmol min^−1^ mg^−1^)	
	Wild-type	W361F
Ginsenoside		
Rb_1_	5082 ± 7.6	3213 ± 10.6
Rb_2_	1643 ± 3.7	9077 ± 15.9
Rc	20.1 ± 0.6	23.3 ± 1.1
Rd	338 ± 1.2	1415 ± 3.8
F_2_	13,130 ± 20.5	13,227 ± 42.3
Compound O	6354 ± 12.8	2031 ± 5.2
Compound Y	4588 ± 10.1	1347 ± 4.9
Compound Mc_1_	1.9 ± 0.2	12.0 ± 0.1
Compound Mc	15.4 ± 0.2	7.2 ± 0.1
Flavanone		
Hesperidin	39.1 ± 0.3	288 ± 0.6
Naringin	233 ± 0.3	35.5 ± 0.2

The kinetic parameters of the wild-type and W361F-variant enzymes for ginsenosides Rb_1_ and Rd were determined (Table 2 and Additional file 1: Figure S2). Although the wild-type enzyme showed higher affinity for the ginsenosides than the W361F variant enzyme, the turnover number and catalytic efficiency of the W361F variant enzyme for ginsenoside Rd were 11- and 3.7-fold higher, respectively, than those of the wild-type enzyme.

**Table 2:** Kinetic parameters of the wild-type and W361F variant enzymes for ginsenosides Rb_1_ and Rd

Enzyme	Substrate	*K*_m_ (mM)	*k*_cat_ (s^−1^)	*k*_cat_/*K*_m_ (s^−1^ mM^−1^)
Wild-type	Rb_1_	0.57 ± 0.01	2257 ± 33	3967 ± 91
	Rd	0.52 ± 0.01	92 ± 1	178 ± 4
W361F	Rb_1_	1.93 ± 0.02	1343 ± 28	697 ± 16
	Rd	1.59 ± 0.02	1049 ± 12	660 ± 11

### Time-course reactions for the hydrolysis of ginsenoside Rb_1_ to compound K by the wild-type and W361F variant enzymes

**Fig. 4:**
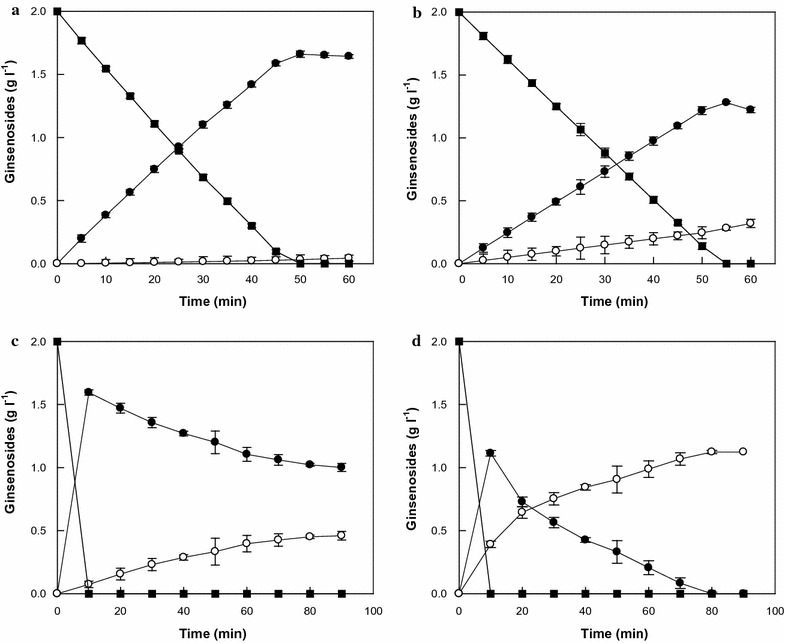
Hydrolysis from ginsenoside Rb_1_ to compound K via Rd and F_2_ by the wild-type and W361F-variant SS-BGLs. Ginsenoside F_2_ was not detected in the hydrolysis reactions because of high specific activity for ginsenoside F_2_. Ginsenoside Rb_1_ (closed square), Rd (closed circle), and compound K (open circle). **a** Using 0.01 mg ml^−1^ wild-type enzyme. **b** Using 0.01 mg ml^−1^ W361F variant enzyme. **c** Using 0.1 mg ml^−1^ wild-type enzyme. **d** Using 0.1 mg ml^−1^ W361F variant enzyme. Data represent the means from three separate experiments, and the error bars represent standard deviations

The reactions for compound K production by the wild-type and W361F variant enzymes were performed with 0.01 mg ml^−1^ enzyme and 2 mg ml^−1^ ginsenoside Rb_1_ for 60 min (Fig. 4a, b). The concentrations of ginsenosides converted by the wild-type and W361F variant enzymes were quantitatively analyzed by an HPLC system during the conversion of ginsenoside Rb_1_ to compound K via Rd (Additional file 1: Figure S3). Ginsenoside F_2_ was not detected in the reactions of either enzyme because the hydrolytic activities for F_2_ were 39- and 9.4-fold higher, respectively, than that for Rd (Table 1). The rate of the hydrolysis of ginsenoside Rb_1_ by the wild-type enzyme was slightly (1.14-fold) faster than that of the variant enzyme. After 60 min, the concentration of compound K produced by the W361F variant enzyme was 0.32 mg ml^−1^, while that produced by the wild-type enzyme was less than 0.04 mg ml^−1^.

The time-course reactions were conducted with a higher enzyme concentration of 0.1 mg ml^−1^. All of the ginsenoside Rb_1_ was completely degraded to Rd and compound K in 10 min by both enzymes (Fig. 4c, d). The W361F variant enzyme converted 2 mg ml^−1^ Rb_1_ to 1.12 mg ml^−1^ compound K after 90 min, with a molar conversion of 100% and a productivity of 843 mg l^−1^ h^−1^, while the wild-type enzyme converted 2 mg ml^−1^ Rb_1_ to 0.46 mg ml^−1^ compound K after 90 min, with a molar conversion of 41% and a productivity of 306 mg l^−1^ h^−1^. The molar conversion and productivity values for the W361F variant enzyme were 2.4- and 2.8-fold higher, respectively, than those of the wild-type enzyme.

## Discussion

V209A variant enzyme was selected with the highest activity by screening of mutant library produced from error-prone PCR. To investigate the effect of the type of the amino acid at position 209 in SS-BGL on the hydrolytic activity for ginsenoside Rd, the amino acid was replaced with other residues. The variant enzymes with only uncharged side chains and no aromatic ring (V209G, V209A, V209T, and V209L) exhibited hydrolytic activity for ginsenoside Rd. With the nonpolar amino acid residues at position 209, the hydrolytic activity for Rd followed the order Ala > Gly > Val > Leu. Thus, the amino acid residue at position 209 is a residue related to Rd-hydrolyzing activity.

The structure of 1GOW was determined without ligand, whereas that of 2CEQ or 2CER was determined as a complex with the smaller molecule glucoimidazole (Mw: 200) or phenethyl-substituted glucoimidazole (Mw: 304) as a ligand than ginsenoside Rd (Mw: 946), respectively. However, there has been no ginsenoside Rd-bound structure. Ginsenoside Rd was not docked to the ligand-bound structure of 2CEQ or 2CER because the enzymes may have a smaller binding pocket. Therefore, ginsenoside Rd was docked only to 1GOW. Numerous forms of docking poses using 1GOW with ginsenoside Rd were generated because ginsenoside Rd has 25 rotatable bonds. Among poses those generated, a docking pose was selected that included the catalytic residues Glu206 and Glu387, and the Val209 residue related to ginsenoside Rd-hydrolyzing activity.

Among the six alanine-substituted variant enzymes, the W361A variant showed the highest activity for ginsenoside Rd hydrolysis. The Trp residue at position 361 of SS-BGL was substituted with charged Lys or Asp, aromatic Phe or Tyr, nonpolar Gly, Ala, or Leu, or polar neutral Thr. The variant enzymes with charged side chains, W361K and W361D, showed lower activity than that of the wild-type enzyme, whereas the variant enzymes with aromatic side chains, W361F and W361Y, exhibited higher activity. The variant enzymes with uncharged side chains, W361G, W361A, and W361T displayed higher activity than that of the wild-type enzyme with the exception of W361L. As the molecular size of the aromatic amino acid side chain decreased, the hydrolytic activity increased (Phe > Tyr > Trp). As a result, the W361F variant enzyme showed the highest activity, and its activity was 2.4- and 1.3-fold higher than V209A and double-site (V209A-W361F) variant enzyme, respectively (data not shown). The binding energy and C-DOCKER interaction energy of the W361F variant enzyme were lower than that of the wild-type enzyme. These results suggest that the W361F variant enzyme formed a more stable and favorable complex with Rd than the wild-type enzyme.

**Fig. 5:**
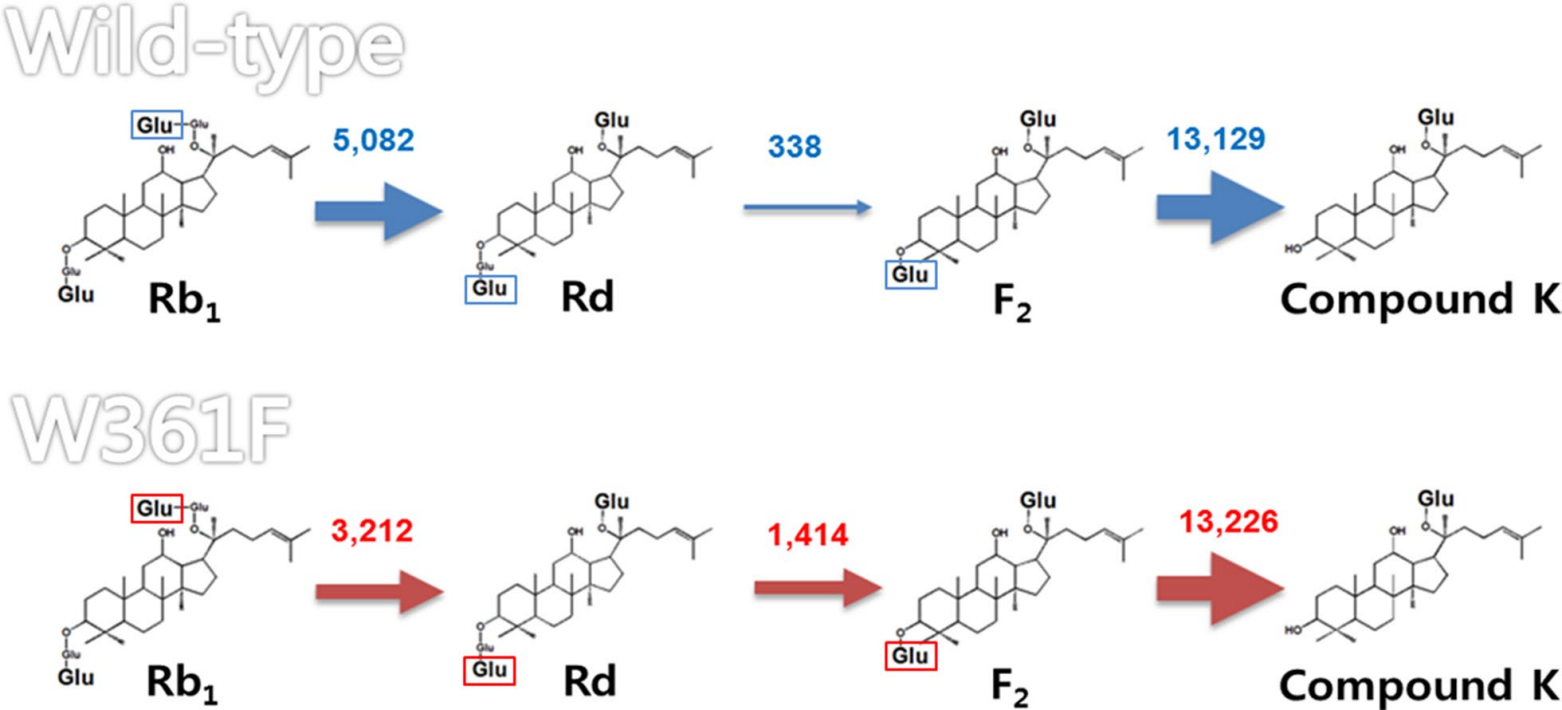
Hydrolytic pathways from ginsenoside Rb_1_ to compound K by the wild-type and W361F variant enzymes. Numbers indicate the specific activity (nmol min^−1^ mg^−1^) for the substrates at each step. The thickness of arrow is depicted in proportion to the specific activity

The W361F variant enzyme exhibited 5.5-, 1.2-, 4.2-, and 6.3-fold higher activity for ginsenosides Rb_2_, Rc, Rd, and compound Mc_1_, respectively, than the wild-type enzyme, whereas the wild-type enzyme showed 1.6-, 3.1-, 3.4-, and 2.1-fold higher activity for ginsenosides Rb_1_, compound O, compound Y, and compound Mc, respectively. The hydrolytic pathway of ginsenoside Rb_1_ to compound K by SS-BGL was Rb_1_→Rd→F_2_→compound K (Fig. 5). Ginsenoside Rd was produced from ginsenoside Rb_1_ by the specific hydrolysis of the outer glucose linked to C-20, while compound K was produced from ginsenoside Rd via F_2_ by the specific hydrolysis of the outer glucose and inner glucose linked to C-3. The wild-type enzyme had 15- and 39-fold higher activity for ginsenoside Rb_1_ and F_2_, respectively, than ginsenoside Rd, indicating that the conversion of Rd to F_2_ is a limiting step for the production of compound K from Rb_1_. The W361F variant SS-BGL exhibited 4.2-fold higher activity for the rate-limiting reaction of Rd to F_2_ than the wild-type enzyme. Thus, the W361F variant enzyme was a suitable enzyme for the improved hydrolysis of ginsenoside Rb_1_ to compound K. The W361F variant completely converted 2 mg ml^−1^ Rb_1_ to 1.12 mg ml^−1^ compound K after 90 min, with a productivity of 843 mg l^−1^ h^−1^ and it was 2.4 higher than that of *Microbacterium esteraromaticum* β-glucosidase, which had the highest previously reported productivity of 460 mg l^−1^ h^−1^ (Quan et al. 2012). Therefore, the W361F variant of SS-BGL was an efficient compound K-producing enzyme using Rb_1_.

SS-BGL has been used for the hydrolysis of glycosides such as stevioside (Nguyen et al. 2016) and isoflavone (Kim et al. 2012), as well as ginsenoside. To investigate the hydrolytic superiority of the variant enzyme for other glycosides, the wild-type and W361F variant enzymes were used for the hydrolysis of two flavanone glycosides, naringin and hesperidin. The specific activity of the W361F variant enzyme for hesperidin was 7.4-fold higher than that of the wild-type enzyme, whereas that of the W361F variant enzyme for naringin was 6.6-fold lower. These results indicate that the alteration of the amino acid at position 361 of SS-BGL results in a significant change in the specificity for flavanone glycosides, and the W361F variant enzyme can be used to increase the production of hesperetin, which has more profound pharmacological activity than hesperidin (Shin et al. 2013).

In conclusion, semi-rational design, the computational analysis of the enzyme structure based on a variant enzyme obtained from a small library, was used to obtain a variant with increased hydrolytic activity for ginsenoside Rd. The obtained W361F exhibited higher activity, higher catalytic efficiency, and predicted lower binding energy than the wild-type enzyme. The variant enzyme completely converted ginsenoside Rb_1_ to compound K with the highest productivity ever reported. The W361F variant enzyme also showed significantly higher activity for the flavanone, hesperidin, than the wild-type enzyme. Therefore, the W361F variant SS-BGL is an efficient glycoside-hydrolyzing enzyme, and semi-rational design is a useful tool for enhancing the hydrolytic activity of specific glycosides linked to ginsenosides. Moreover, to the best of our knowledge, this is the first attempt to improve the hydrolytic activity of an enzyme that converts ginsenosides using protein engineering.

## Additional file

**Additional file 1: Figure S1.** Effects of **a** pH and **b** temperature on the activity of the wild-type (closed circle) and W361F (open circle) variant enzymes. Data represent the means from three separate experiments, and the error bars represent standard deviations. **Figure S2.** Hanes-Woolf plots **a** using wild-type enzyme and ginsenoside Rb_1_, **b** using wild-type enzyme and ginsenoside Rd, **c** using W361F variant enzyme and ginsenoside Rb_1_, and **d** using W361F variant enzyme and ginsenoside Rd. **Figure S3.** HPLC profiles for the conversion of ginsenoside Rb_1_ to compound K via ginsenoside Rd by the wild-type and W361F variant enzymes. The retention times for ginsenoside Rb_1_, Rd, and compound K were 10.2, 13.4, and 29.8 min, respectively. **Table S1.** Abbreviations for ginsenosides. **Table S2.** Relative activities of the wild-type and W361F variant enzymes for aryl-glycosides.

## Abbreviations

PPD: protopanaxadiol
PPT: protopanaxatriol
SS-BGL: β-glycosidase from *Sulfolobus solfataricus*
LB: Luria–Bertani
IPTG: isopropyl-β-d-thiogalactopyranoside
SDM: site-directed mutagenesis
pNP: p-nitrophenol
