# Supplementary material for: Improved conversion of ginsenoside Rb1 to compound K by semi-rational design of Sulfolobus solfataricus β-glycosidase
Source: AMB Express. 2017 Oct 4;7:186. doi: 10.1186/s13568-017-0487-x (PMC5628084; doi:10.1186/s13568-017-0487-x)
Supplement: Supplementary file 1 — Additional file 1: Figure S1. Effects of a pH and b temperature on the activity of the wild-type (closed circle) and W361F (open circle) variant enzymes. Data represent the means from three separate experiments, and the error bars represent standard deviations. Figure S2. Hanes-Woolf plots a using wild-type enzyme and ginsenoside Rb1, b using wild-type enzyme and ginsenoside Rd, c using W361F variant enzyme and ginsenoside Rb1, and d using W361F variant enzyme and ginsenoside Rd. Figure S3. HPLC profiles for the conversion of ginsenoside Rb1 to compound K via ginsenoside Rd by the wild-type and W361F variant enzymes. The retention times for ginsenoside Rb1, Rd, and compound K were 10.2, 13.4, and 29.8 min, respectively. Table S1. Abbreviations for ginsenosides. Table S2. Relative activities of the wild-type and W361F variant enzymes for aryl-glycosides. [file 13568_2017_487_MOESM1_ESM.docx]

**Additional file**

**AMB Express**

Improved conversion of ginsenoside Rb_1_ to compound K by semi-rational design of *Sulfolobus solfataricus* β-glycosidase

Kyung-Chul Shin ∙ Hye-Yeon Choi ∙ Min-Ju Seo ∙ Deok-Kun Oh

Corresponding author: D.-K. Oh

Department of Bioscience and Biotechnology, Konkuk University, Seoul 05029, Republic of Korea, E-mail: [deokkun@konkuk.ac.kr](mailto:deokkun@konkuk.ac.kr), Tel.: +82-2-454-3118; Fax: +82-2-444-5518

**a**

**b**

**Figure S1** Effects of **a** pH and **b** temperature on the activity of the wild-type (closed circle) and W361F (open circle) variant enzymes. Data represent the means from three separate experiments, and the error bars represent standard deviations.

**a b**

**c b**

**Figure S2** Hanes-Woolf plots **a** using wild-type enzyme and ginsenoside Rb_1_, **b** using wild-type enzyme and ginsenoside Rd, **c** using W361F variant enzyme and ginsenoside Rb_1_, and **d** using W361F variant enzyme and ginsenoside Rd.
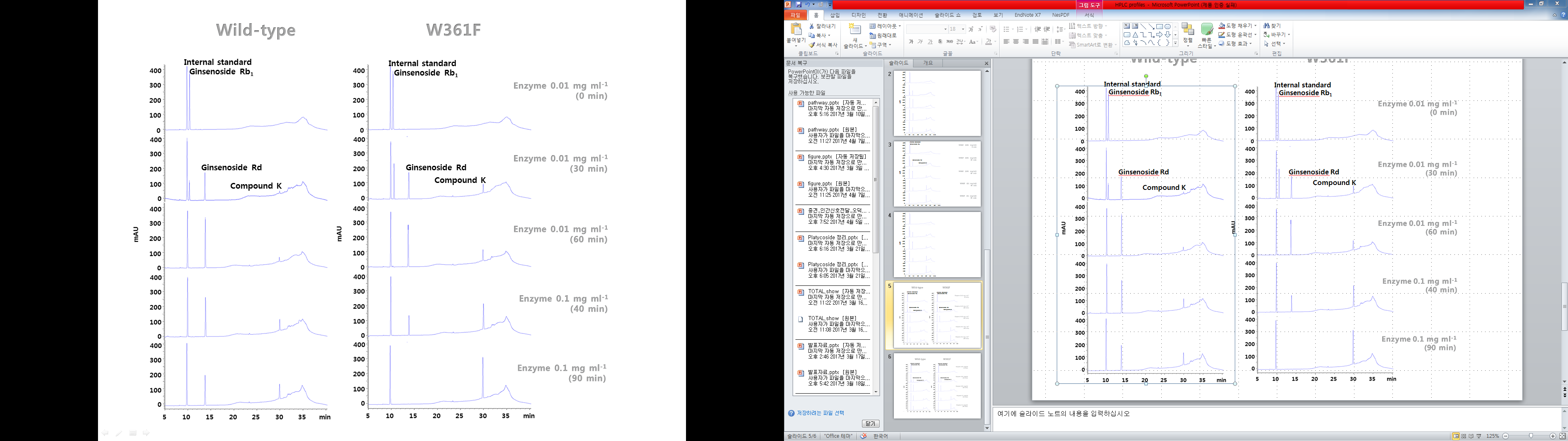


**Figure S3**  HPLC profiles for the conversion of ginsenoside Rb_1_ to compound K via ginsenoside Rd by the wild-type and W361F variant enzymes. The retention times for ginsenoside Rb_1_, Rd, and compound K were 10.2, 13.4, and 29.8 min, respectively.

**Table S1** Abbreviations for ginsenosides

| Abbreviation | Full name |
| --- | --- |
| Rb_1_ | 3-O-[β-d-glucopyranosyl-(1→2)-β-d-glucopyranosyl]-20-O-[β-d-glucopyranosyl-(1→6-β-d-glucopyranosyl]-20(S)-protopanaxadiol |
| Rb_2_ | 3-O-[β-d-glucopyranosyl-(1→2)-β-d-glucopyranosyl]-20-O-[α-l-arabinopyranosyl-(1→6-β-d-glucopyranosyl]-20(S)-protopanaxadiol |
| Rc | 3-O-[β-d-glucopyranosyl-(1→2)-β-d-glucopyranosyl]-20-O-[α-l-arabinofuranosyl-(1→6-β-d-glucopyranosyl]-20(S)-protopanaxadiol |
| Rd | 3-O-[β-d-glucopyranosyl-(1→2)-β-d-glucopyranosyl]-20-O-β-d-glucopyranosyl-20(S)-protopanaxadiol |
| Rg_3_ | 3-O-[β-d-glucopyranosyl-(1→2)-β-d-glucopyranosyl]-20(S)-protopanaxadiol |
| Gypenoside XVII | 3-O-β-d-glucopyranosyl-20-O-[β-d-glucopyranosyl-(1→6-β-d-glucopyranosyl]-20(S)-protopanaxadiol |
| Compound O | 3-O-β-d-glucopyranosyl-20-O-[α-l-arabinopyranosyl-(1→6-β-d-glucopyranosyl]-20(S)-protopanaxadiol |
| Compound Mc_1_ | 3-O-β-d-glucopyranosyl-20-O-[α-l-arabinofuranosyl-(1→6-β-d-glucopyranosyl]-20(S)-protopanaxadiol |
| F_2_ | 3-O-β-d-glucopyranosyl-20-O-β-d-glucopyranosyl-20(S)-protopanaxadiol |
| Rh_2_ | 3-O-β-d-glucopyranosyl-20(S)-protopanaxadiol |
| Gypenoside LXXV | 20-O-[β-d-glucopyranosyl-(1→6-β-d-glucopyranosyl]-20(S)-protopanaxadiol |
| Compound Y | 20-O-[α-l-arabinofuranosyl-(1→6-β-d-glucopyranosyl]-20(S)-protopanaxadiol |
| Compound Mc | 20-O-[α-l-arabinofuranosyl-(1→6-β-d-glucopyranosyl]-20(S)-protopanaxadiol |
| Compound K | 20-O-β-d-glucopyranosyl-20(S)-protopanaxadiol |
| APPD | 20(S)-protopanaxadiol |
| R_1_ | 6-O-[β-d-xylopyranosyl-(1→2)-β-d-glucopyranosyl]-20-O-β-d-glucopyranosyl-20(S)-protopanaxatriol |
| Re | 6-O-[α-l-xylopyranosyl-(1→2)-β-d-glucopyranosyl]-20-O-β-d-glucopyranosyl-20(S)-protopanaxatriol |
| Rf | 6-O-[β-d-glucopyranosyl -(1→2)-β-d-glucopyranosyl]-20-O-β-d-glucopyranosyl-20(S)-protopanaxatriol |
| Rg_1_ | 6-O-β-d-glucopyranosyl-20-O-β-d-glucopyranosyl-20(S)-protopanaxatriol |
| F_1_ | 20-O-β-d-glucopyranosyl-20(S)-protopanaxatriol |
| R_2_ | 6-O-[β-d-xylopyranosyl-(1→2)-β-d-glucopyranosyl]-20(S)-protopanaxatriol |
| Rg_2_ | 6-O-[α-l-xylopyranosyl-(1→2)-β-d-glucopyranosyl]-20(S)-protopanaxatriol |
| Rh_1_ | 6-O-β-d-glucopyranosyl-20(S)-protopanaxatriol |
| APPT | 20(S)-protopanaxatriol |

**Table S2** Relative activities of the wild-type and W361F variant enzymes for aryl-glycosides

| Substrate | Relative activity (%) | |
| --- | --- | --- |
|  | Wild-type | W361F |
| pNP-*β*-d-Glucopyranoside | 100.0 ± 0.5 | 80.6 ± 0.2 |
| pNP-*α*-d-Glucopyranoside | ND | ND |
| oNP-β-d-Glucopyranoside | 44.4 ± 0.3 | 26.8 ± 1.1 |
| pNP-β-d-Galactopyranoside | 61.9 ± 0.2 | 33.1 ± 0.1 |
| pNP-α-d-Galactopyranoside | ND | ND |
| oNP-β-d-Galactopyranoside | 45.4 ± 0.1 | 28.6 ± 0.3 |
| pNP-β-d-Xylopyranoside | 9.5 ± 0.4 | 7.1 ± 0.1 |
| oNP-β-d-Xylopyranoside | 3.9 ± 0.2 | 5.1 ± 0.1 |
| pNP-β-d-Mannopyranoside | 8.5 ± 0.2 | 4.9 ± 0.2 |
| pNP-α-l-Rhamnopyranoside | ND | ND |
| pNP-α-l-Arabinopyranoside | 10.2 ± 0.6 | 8.6 ± 0.3 |
| pNP-α-l-Arabinofuranoside | 0.7 ± 0.2 | 0.4 ± 0.1 |

ND, not detected
